# Application of a catalytic oxidation method for the simultaneous determination of total organic carbon and total nitrogen in marine sediments and soils

**DOI:** 10.1371/journal.pone.0252308

**Published:** 2021-06-04

**Authors:** Pavlos Avramidis, Vlasoula Bekiari

**Affiliations:** 1 Department of Geology, University of Patras, Rio-Patras, Greece; 2 Department of Crop Science, University of Patras, Messolonghi, Greece; Universiti Malaysia Pahang, VIET NAM

## Abstract

This study presents the application of a simultaneous method for the determination of total organic carbon (TOC) and total nitrogen (TN) in marine sediments and soils, using a data set of 206 samples collected from coastal lagoonal/marine sedimentary environments and certified reference materials (CRMs). TOC and TN were determined using the high temperature (720°C) catalytic (Pt/Al_2_O_3_) oxidation method and the detection of TOC and TN was performed using an infrared or a chemiluminescence detector, respectively. Results from the abovementioned TOC method were compared with the results from the widely used Wakley-Black titration method, while TN results with these from elemental analysis. Analytical quality control experiments were performed using CRM samples. Method characteristics such as range of measurement, calibration, method detection limit (MDL), limit of quantification (LOQ), repeatability and reproducibility, were calculated. The precision and the accuracy of the methods are also discussed. Comparison of the two TOC methods of 206 data set yields a regression line of correlation coefficient with R^2^ = 0.985. Additional different levels of TOC concentrations of low <1%, moderate 1–5% and high 5–40% level were examined indicating very good correlations. The lowest correlation coefficient was observed in low concentrations TOC<1% (R^2^ 0.825), mainly as a result of the limitation of titration method. The evaluation of TN results indicated that the catalytic oxidation method and the elemental analysishave a significant good correlation with R^2^ = 0.977. The results of precision and accuracy, as well as the calculated MDL and LOQ show that this is a reliable method. Moreover, it requires a small amount of the analyzed sample and the total analysis time is 10 min. Therefore, it can be easily applied for the fast and precise simultaneous determination of TOC and TN in sediment and soil samples.

## Introduction

Total organic carbon (TOC) and total nitrogen (TN) are important parameters for assessing the environmental status of terrestrial and aquatic ecosystems. The organic carbon and nitrogen in soils and sediments are mainly coming from decomposition of the animals and plants. Moreover, anthropogenic sources such as chemical impurities, fertilizers or organic-rich wastes, enrich the ecosystems with organic carbon and nitrogen. Both TOC and TN are often used as an index of the available amount of food to benthic animals. Additionally, both are used in aquatic systems as an important index of the organic load settling to the bottom sediments from the water column. In these systems, the presence of TOC and TN affect the faunal communities as it is crucial for the primary production which is a key issue for the eutrophication status.

The similar chemical nature of the organic matter of sediments and soils to non-polar organic pollutants allows their preferential distribution in them [1]. The organic matter in sediments do not allow contaminants that form ions such as phenols or metals to partition strongly into the organic fraction due to the absence of electrostatic interactions, while non-pollar organic contaminants are more preferrable due to hydrophobic interactions. So, the sediments and soils concentration of the organic carbon is very well correlated with the type of the organic contaminants and for this reason, can be used as a tool for the estimation of the level and the nature of contamination [2]. Therefore, methods of sediments and soils organic carbon normalization, which expresses the concentration of these chemicals in the organic carbon fraction of sediments and not in the overall dry weight, have been developed and reviewed by Michelsen [3].

Another important use of TOC/TN ratio in sediments is to distinguish the marine (algae) or terrestrial (land plants) sources of organic matter as well as aerobic/anoxic and sulfate reduction conditions [4–6] or as palaeoenvironmental proxy [7, 8]. A marine depositional environment typically has atomic C/N ratios between 4 and 10, whereas a terrestrial environment has C/N ratios of >20 [9]. These ratios have been used widely as biomarkers for the reconstruction of sedimentological depositional environments and the environmental changes of the past. Soil is one of the most important and largest carbon reservoir in the world. Soil total carbon (TC), soil organic matter (SOM) and soil total nitrogen (STN) are the major parameters for soil fertility and productivity characterization, and the main indices of soil quality.

For all the above reasons several methods have been developed for the qualitative, semi-quantitative and quantitative estimation and definition of TOC [10]. Some of them use modern methods of instrumental chemical analysis and others are inexpensive chemical methods based on classical analytical techniques. As a qualitative method, we refer to nuclear magnetic resonance (NMR) which provides rapid and inexpensive information about the carbon forms in soil and sediments [11]. The two main semi-quantitative methods are the loss on ignition (LOI) [12] and the hydrogen peroxide digestion [13, 14]. In the quantitative instrumental methods are included the LECO (carbon analysis) method [15], the RockEval pyrolysis [16], the loss on ignition (LOI) [17–19] and the combustion-infrared method [20, 21]. Concerning classical analysis the most inexpensive and widely used method is the Wakley-Black wet oxidation—titration method [22]. This method has been validated and compared with LECO by Gaudette et al., [23] and later by Beaudoin [24] and with the combustion-infrared method by Avramidis et al., [21]. As this research subject is of high scientific interest, a comparison of different analytical methods for determining the organic and inorganic carbon content, using lake sediments and soils have been recently reported by Wang et al. [20], while a new method has been proposed by Xu et al. [25] for organic rich marine sediments.

The high temperature catalytic oxidation method for the determination of TOC, presented in this study, has the main advantage of the simultaneous determination of TN. This is achieved by coupling a chemiluminescence detector for detection of NO_x_ gases [26]. As the determination of TOC and TN has a significant importance in the studies of a variety of environmental systems [27, 28], development of novel environmentally friendly materials, water purification technologies [29–31] and food microbiology [32], the above mentioned procedure has been receiving an increasing scientific interest during the last years. It should be noted that the main advantages of this method are speed, accuracy and no production of hazardous wastes [33–35].

In the present work, TOC and TN were simultaneously measured in sediment and soil samples, by the catalytic oxidation method. The catalyst used for the oxidation was Pt/Al_2_O_3_, while the applied temperature was 720°C [21, 35]. Concerning this particular method of analysis, as it described in our previous works [21, 35], it is applicable for different kinds of liquid or solid samples. Briefly, the contained organic carbon and nitrogen are oxidized and converted to carbon dioxide and nitrogen oxides respectively. The produced gases are then detected with an infrared detector in the case of carbon dioxide or a chemiluminescence detector for nitrogen oxides [21, 35].

With regard to the determination of TOC, the specific method first determines total carbon (TC), while inorganic carbon (IC) is measured and a different stage by acidification of the sample which results to its conversion to carbon dioxide. TOC is then calculated as TOC = TC-IC [21, 35]. For both TOC and TN measurements, we used CRMs for the estimation of methods characteristics such as range of measurement, calibration and method validation [21, 35–37]. Additionally, a dataset of sediments which are coming from different Greek coastal environments (lagoonal and marine) and soils were studied. In the case of TOC measurements in sediments the results were compared with wet oxidation—titration method of Wakley and Black [22], while the results for the TN determination were compared with the results from elemental analysis.

The novelty of this work is the development and certification of an instrumental, low laboratory time consume chemical analysis method for the simultaneous determination of TOC and TN in environmental solid samples. This method can have an important application where the above chemical analyses are required to determine the quality of the environment as it is applicable to all types of samples and at concentration levels where limitations from conventional chemical analysis methods exist.

## Materials and methods

### Catalytic oxidation method for the simultaneous determination of TOC and TN in sediments and soils

Ultrapure water, purified by using a TKA Smart2Pure apparatus, with TOC less than 5 ppb was used for the preparation of all solutions. Simultaneous analyses of TOC and TN were carried out using a Shimadzu TOC analyzer (TOC-VCSH) coupled to a chemiluminescence detector (TNM-1 TN unit), creating a simultaneous analysis system. Concerning these analyses, commercially available reagent grade stock acid solutions were used for the preparation of the diluted 2 M hydrochloric acid solution used in this method. The carrier, purging and reactive gas was synthetic air, CO_2_ free, containing less than 1 ppm hydrocarbon.

The stock solutions for the calibration measurements for the organic and the inorganic carbon, as well as for the total nitrogen, were prepared according to the procedure described in detail by Bekiari and Avramidis elsewhere [35]. Briefly, anhydrous primary-standard-grade potassium biphthalate (C_8_H_5_KO_4_, Merck) was used for the preparation of the 1 mg/mL organic carbon stock solution, while anhydrous sodium carbonate (Na_2_CO_3_, Merck) and anhydrous sodium bicarbonate (NaHCO_3_, Merck) were used for the preparation of the 1 mg/mL inorganic stock solution. The 1 mg/mL nitrogen stock solution was prepared by using anhydrous special reagent grade potassium nitrate (KNO_3_, Merck). Proper dilution with ultra pure water of the above stock solutions was done for the preparation of all standard solutions used for calibration or quality control of TC, IC and TN. For all the studied parameters the concentration range for calibration was 0–100 mg/L and for this purpose ten standard solutions were prepared with the following concentrations: 0.1 mg/L, 0.2 mg/L, 0.5 mg/L, 1 mg/L, 2 mg/L, 5 mg/L, 10 mg/L, 20 mg/l, 50 mg/L and 100 mg/L.

**Fig 1:**
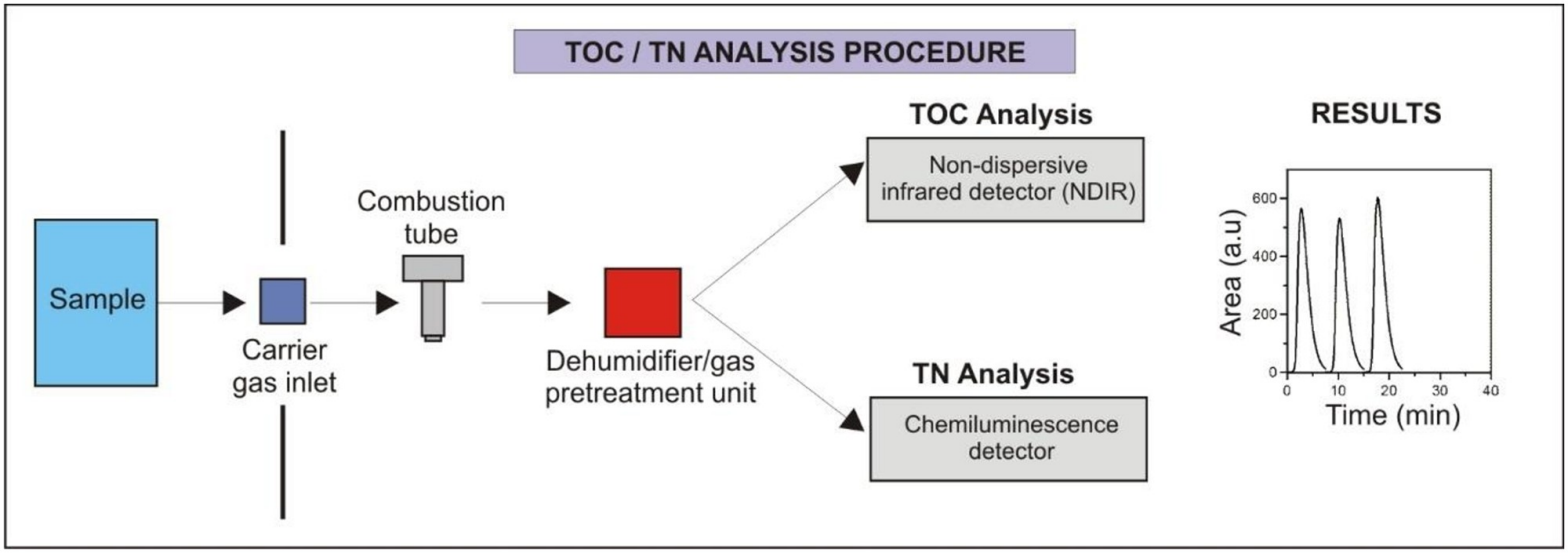
Schematic presentation of TOC and TN analytical procedure applying high temperature (720°C) catalytic combustion (Pt/Al_2_O_3_) oxidation method.

As it is described in our previous work [35], the principle for the TOC method is that the sample is heated with the Pt/Al_2_O_3_ oxidative catalyst. All the contained carbon (organic and inorganic) is oxidized to carbon dioxide and water. The produced gas is transferred in the carrier gas (purified air) and is quantified by an infrared analyzer (NDIR analyzer). Inorganic carbon (IC) is quantified in a next step with the same detector by acidification of the sample with HCl acid at pH<3. TOC is obtained by difference (TOC = TC-IC). In the case of TN determination, all the contained nitrogen is oxidized to nitric oxide (NO) and it is detected by a chemiluminescence method [35]. A schematic presentation of the analytical procedure concerning TOC-TN simultaneous analysis is presented in Fig 1. Both methods were calibrated for the determination of linear response and validated for the calibration functions by using (CRM/TOC-TN). For the measurement of soils and sediments, 0.200 g of pulverized sample is transferred in an Erlenmeyer flask and is suspended in a 200 mL diluted hydrochloric acid solution (HCl 0.22 mol/L). The suspension is dispersed and homogenized using a dispersion drive for three minutes at a speed of 17000 to 18000 rpm.

### Wet oxidation-titration method for the determination of TOC in sediments and soils

All the reagents for the wet oxidation-titration method for the TOC determination in solid samples were purchased from Aldrich and were of analytical grade. This method [23] uses potassium dichromate (K_2_Cr_2_O_7_) and concentrated H_2_SO_4_ for exothermic heating and oxidation of the contained organic carbon. Then, the excess of dichromate is titrated with 0.5 N ferrous ammonium sulphate (NH_4_)_2_Fe(SO_4_)_2_·6H_2_O) solution to a sharp one drop endpoint. In this procedure, 10 mL of 1 N K_2_Cr_2_O_7_ solution is added to 0.2 to 0.5 g dried sample in a 500 mL Erlenmeyer flask and are mixed by swirling. Then, twenty (20) mL of concentrated H_2_SO_4_ are added and mixed gently. The mixture is allowed to react for 30 minutes and after is diluted to 200 mL volume with distilled water and 10 mL of 85% H_3_PO_4_. 0.2 g NaF and 15 drops of diphenylamine indicator are added. The diphenylamine indicator is prepared by dissolving 0.5 g of reagent grade diphenylamine to 20 mL of ultrapure water and 100 mL of concentrated H_2_SO_4_. The solution is back titrated with ferrous ammonium sulfate solution 0.5 N. The results of the analysis are calculated by the following equation:

$$\%\;TOC=10-(1-\frac{T}{S})(1.0N\;0.003)(\frac{100}{W})$$ (1)

Where: T = sample titration, mL ferrous ammonium sulfate solution

S = standardization blank titration, mL ferrous ammonium sulfate solution

0.003 = 12/4000 = meq weight of carbon

1.0 N = normality of K_2_Cr_2_O7

10 = volume of K_2_Cr_2_O_7_ in mL

W = weight of sediment sample in grams

### Elemental analysis for the determination of TN in sediments and soils

TN determination with elemental analysis was performed by using Carlo Erba EA 1108 Elemental Analyzer. This instrument analyses ground solid samples for the quantification of total carbon, organic carbon, black carbon, nitrogen, sulphur and hydrogen. The chromatographic signals are calibrated using pre-analyzed standards and the elemental concentrations of carbon and nitrogen are given in weight percent. Equipment operation, data storage, as well as post-run analysis were done by using Eager 200 software. The method uses flash combustion at 1020°C of dried, ground samples which have been weighed into tin capsules. Samples are placed inside the autosampler which is purged with helium, an inert gas. The helium carrier gas takes the gases produced from the flash combustion through the combustion reactor which contains the oxidation catalysts of tungsten trioxide (WO_3_), a copper reducer and platinized alumina. Firstly, all the contained nitrogen is oxidized to nitrogen oxides (NO_x_). Then, the helium carrier gas transfers the gases into the reduction column containing copper wire as catalyst. The reduction column reduces the nitrogen oxides to N_2_ gas. The resulting mixture of gases is carried by the helium carrier gas through a chromatographic column (2-m-long packed column Poropak Q/S 50/80 mesh) which separates the produced gases. The separated gases are detected by a thermal conductivity detector (TCD).

### Description of the analyzed samples

For this study, the samples were collected from three coastal sedimentary environments of western Greece, Gialova, Prokopos and Aetoliko lagoons [38–40] and Keri coastal Lake [41] as well as from marine depositional environments of Ionian [42] and the Aegean Sea. TOC content for a total number of 206 samples was determined by using the catalytic oxidation method, as well as the wet oxidation-titration. Concerning TN determination, 43 from the above mentioned samples were analyzed by using the catalytic oxidation method as well as the elemental analysis. Validation of TOC and TN methods was performed by the analysis of two CRMs. The CRMs used for TOC were IRMM-443-7, Eurosoil 7 (LGC Standards) (TOC: 5.62%) and AgroMat—Compost CP-1 (SCP Science) (TOC 28%), while for TN were IRMM-443-7, Eurosoil 7 (TN: 0.48%) and C4A-UP (Carlo Erba Reagents) (TN: 6.31%).

## Results

### Validation of simultaneous TOC-TN determination-Data analysis

#### Calibration-linearity -range of measurements

**Fig 2:**
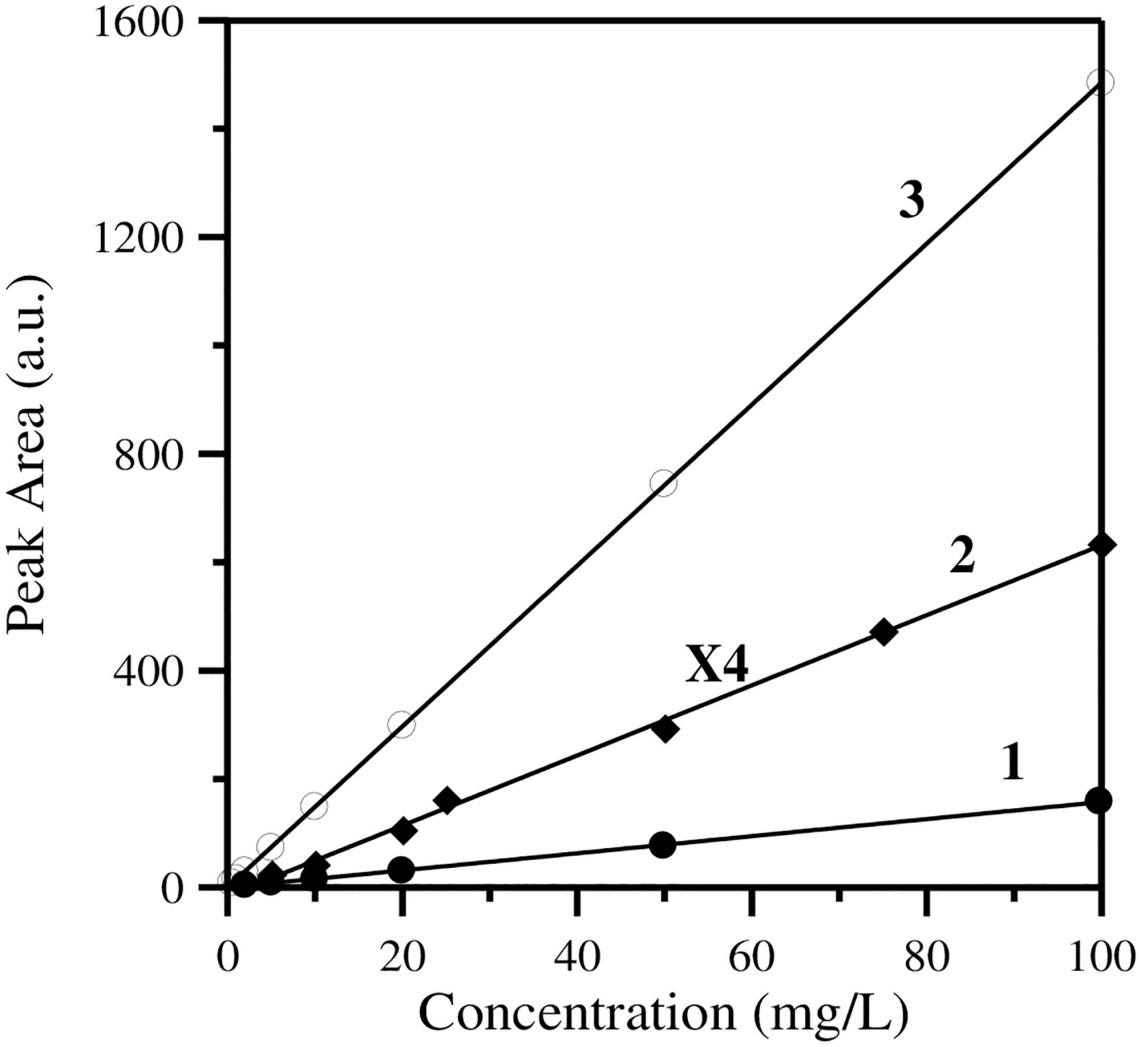
Representative calibration curves for TC (1), IC (2) and TN (3).

As it is extensively presented in our previous publication [35], the calibration curves for all parameters were constructed by measuring ten standard solutions with concentrations covering the full range of the measurement area (0.1 mg/L, 0.2 mg/L, 0.5 mg/L, 1 mg/L, 2 mg/L, 5 mg/L, 10 mg/L, 20 mg/l, 50 mg/L and 100 mg/L). The calibration curves were constructed by plotting peak area versus concentration. Linearity is one of the most important characteristics of calibration curves in instrumental analysis. The characteristic calibration curves used for the TC, IC and TN analyses in this work are shown in Fig 2 and as we can see in all cases we have satisfactory linear graphs (R^2^≥0.995). The range of measurements for the solid samples was dictated by the range of the aqueous samples that could be analyzed by this system [35] and are in both cases (TOC and TN) from 0.05% till 100%.

#### Method detection limit (MDL)-limit of quantification (LOQ)

The method detection limit (MDL) is defined as the lowest concentration level that can be determined and it is statistically different from the blank. The limit of quantification (LOQ) is defined as the concentration level which can be securely quantified taking into account the standard deviation of the measurements. [36]. In our studies for TOC and TN in solid samples analyses these values were dictated by the respective limits in the aqueous samples that could be analyzed by this system [37]. They are estimated at 0.015% (MDL) and 0.030% (LOQ) for TOC, while for TN the same parameters are 0.010% and 0.020%, respectively.

#### Methods precision and accuracy

The precision of a method is a measure of the random error of repeated measurements under the same conditions and it is expressed as the standard deviation of repeatability (S_r_) and reproducibility (S_R_). In this study, repeatability is determined by fifteen-sample repeat analysis of each CRM on the same day by the same analyst. In terms of the estimation of reproducibility the experimental data were obtained by using the fifteen measurements performed of the same CRM samples. The measurements were performed by two different analyzers over a period of two months. Repeatability and reproducibility data are presented in Table 1. Method accuracy was checked by comparing the mean value of our experimental results (X_mean_) with the certified values of the CRMs (certified concentration) as they are presented in Table 1. For both parameters (TOC and TN) the measured values have a difference less than 0.4%. Finally, for all the CRMs the TOC and the TN results obtained from the catalytic oxidation method are compared with the results obtained from the wet-oxidation-titration method and the elemental analysis method, respectively. This comparison is presented in Table 2.

**Table 1:** TOC and TN precision and accuracy data.

Parameter	Certified	Repeatability		Reproducibility	
	Concentration %	X_mean_ (n = 20)	S_r_	X_mean_ (n = 20)	S_R_
TOC	5.62%.	5.64%	0.62	5.61%	0.59
TOC	28%	29%	3.61	29%	3.79
TN	0.48%	0.46%	0.03	0.47%	0.03
TN	6,31%	6.34%	0.71	6.39%	0.77

**Table 2:** TOC and TN values for the CRM by the two different determination techniques.

Parameter	Certified Concentration	Concentration from Oxidation Infrared Method	Concentration from Wet Oxidation-Titration Method
TOC	5.62%.	5.60%	4.94%
TOC	28%	29%	21%
Parameter	Certified Concentration	Concentration from Oxidation Chemiluminescence Method	Concentration from Elemental analysis Method
TN	0.48%.	0.45%	0.47%
TN	6.31%	6.34%	6.32%

#### Total organic carbon TOC-comparison of the catalytic oxidation method with the wet oxidation-titration method

**Fig 3:**
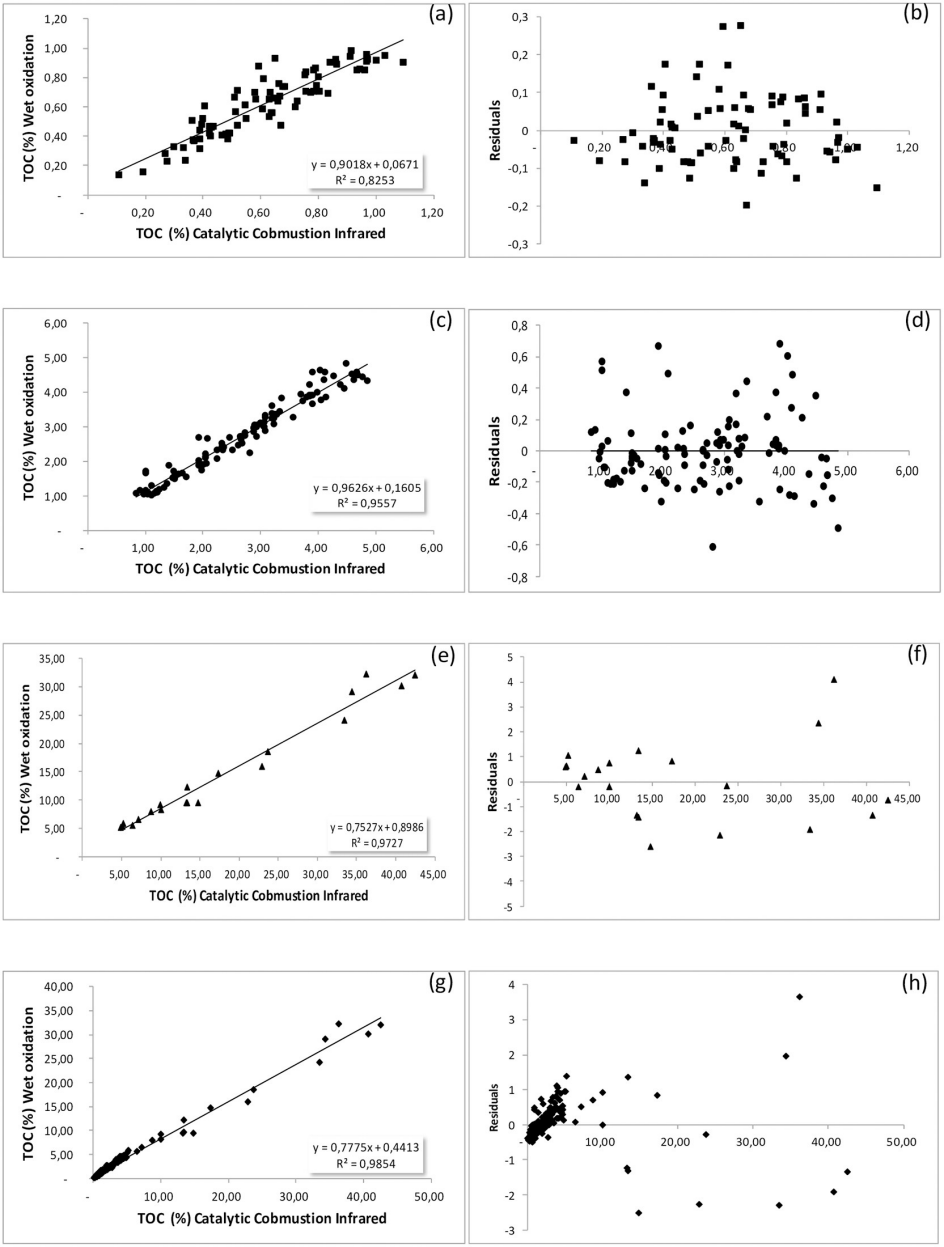
Scatter diagrams, best fitted lines and residual diagrams for: (a)(b) low TOC concentration <1%, (c)(d) moderate TOC concentration 1–5%, (e)(f) high TOC concentration >5% and (g)(h) all samples.

Our sediments data set was separated into three main examined categories based on TOC concentration: (a) low <1%, (b) moderate 1–5% and (c) high >5%. The results of the analyzed samples by wet oxidation—titration and the oxidation method were studied based on regression analysis and scatter plot diagrams with regression lines and residual plots were constructed (Fig 3). The results of the regression analysis are shown in Table 3. In our study, all comparisons were not taking into account the correction factor of Wakley method [22], which Gaudette et al., applied for the unrecovered carbon [23].

**Table 3:** The results of the regression analysis, between TOC measurements from the catalytic oxidation method and the wet oxidation/titration method and between TN measurements from the catalytic oxidation method and the elemental analysis.

Range of measurements	Number of samples	Intercept		Slope		R-square
Concentration Area		Value	Error	Value	Error	
TOC Low <1%	79	0.067	0.031	0.901	0.047	0.825
TOC Moderate1-5%	107	0.160	0.059	0.962	0.020	0.955
TOC High >5%	20	0.898	0.047	0.752	0.029	0.972
TOC All samples	206	0.441	0.047	0.777	0.006	0.985
TN All samples	43	-0.012	0.009	0.984	0.008	0.977

The comparison of the two different methods with the regression analysis indicates a very good correlation, with regression coefficients greater than 0.825 in the three concentration ranges. More specifically, in low concentrations area (<1%) the intercept point is -0.067 and the regression coefficient R^2^ = 0.825 for 79 analyzed samples and significance level p<0.001 (Fig 3a and 3b and Table 3). In moderate concentrations area (1–5%) the intercept point is 0.160, the regression coefficient R^2^ = 0.955 for 107 analyzed samples and significance level p<0.001(Fig 3c and 3d and Table 3). In high concentrations (>5%, 20 analyzed samples) the intercept point is 0.898 and the regression coefficient R^2^ = 0.972 with significance level p<0.001(Fig 3e and 3f and Table 3). Finally, the comparison of the whole samples (total samples 206) indicates a pronounced linear trend, with a high correlation coefficient of the two methods with R^2^ = 0.988 (p<0.001) and intercept 0.441 (Fig 3g and 3h and Table 3).

#### Total nitrogen TN-comparison of the catalytic oxidation method with the elemental analysis method

**Fig 4:**
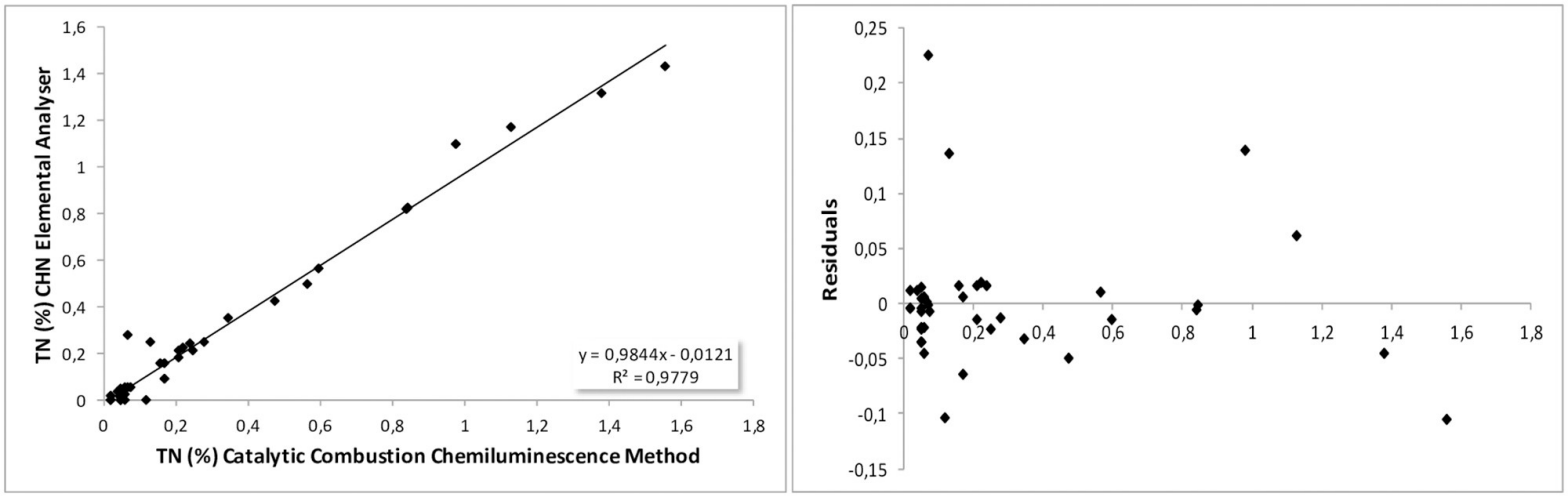
(a) Scatter diagram and best-fitted line and (b) residual diagram for TN comparison.

The TN content of 43 samples, determined by the oxidation method and the elemental analysis, were compared with regression statistics using scatter plot diagrams, regression lines and residual plots. The examined data set consists of sediments and soils with total nitrogen content <2%. A high correlation was revealed with a regression coefficient of R^2^ = 0.977 and intercept value of -0.012 with significance level p<0.001 (Fig 4a and 4b and Table 3).

## Discussion

In the present work, two fully validated methods for simultaneous determination of TOC and TN in solid samples were developed with total analysis time 10min. The C/N ratio is considered as an indicator that determines the decomposition of soil organic matter, while nitrogen (N) is a key component of the nutrient cycle in all ecosystems, as its concentration characterizes the balanced quality of water and soil. Excessive amounts of N not used by living organisms is harmful to the environment [43].

The C/N ratio in the sediments represents the amount of total nitrogen (TN) per unit of total carbon content (TC) and is considered an indicator that determines the rate of mineralization, immobilization and nitrification in the soil. In general, ratios above 25 indicate slow decomposition rates, while ratios below 25 represent faster decomposition rates of organic matter [44]. So, the fast and accurate determination of these parameters is crucial for many scientists [45, 46]. This study demonstrates that these methods of measuring TOC and TN simultaneously are suitable for the determination of organic charge and nitrogen in sediments and soils as well as environmental solid samples. The values of TOC and TN were highly correlated with the respective values obtained from the widely used wet oxidation titration method and the elemental analysis method, respectively.

The determination of TOC for sediment and soil samples and two certified reference materials (CRM) (208 samples) by the catalytic oxidation method and the wet oxidation-titration method showed a high correlation coefficient (R^2^ = 0.988) between the two different methods. Additionally, in the case in the case of TN the measurement of 43 aquatic sediment samples and two CRMs, indicated also a very good correlation (R^2^>0.977) between the catalytic oxidation method and the elemental analysis method. Moreover, the validation analysis indicated that both methods are characterized by accuracy and precision.

At this point it should be noted that in addition to the two main advantages of the specific methods of analysis, which are the simultaneous determination and the low laboratory time consume, several improvements concerning analytical and environmental aspects are also important. In Table 4 we tried a brief comparison of the most widely used analytical methods for the determination of TOC and TN with the catalytic oxidation method.

**Table 4:** Comparison of the most widely used analytical methods for the determination of TOC and TN with the catalytic oxidation method.

Analytical Method	Description	Application field	Main Advantages/Disadvantages
Wakley-Black wet oxidation—titration method Ref. [22–24]	Classical analytical technique, Quantitative method	TOC analysis of soils and marine sediments.	Low cost, no need for expensive equipment /Not applicable to soils containing significant amounts of inorganic carbon, underestimation of TOC in samples with very low or high organic content, use of hazardous chemicals
Hydrogen peroxide digestion Ref. [13, 14]	Instrumental analysis, Semi-quantitative method	TOC and TN analysis in marine sediments and farm-scale samples	Low cost, simple and safe /Underestimation of the organic content and limited applicability for TN in clay minerals
Nuclear Magnetic Resonance (NMR) Ref. [11]	Instrumental analysis, Qualitative method	Characterization of soil organic matter and humification	No extraction of organic matter and so no use of chemicals is needed /Expensive, time consuming
Loss on Ignition Ref. [12, 17, 19]	Instrumental analysis, Semi-quantitative method	TOC analysis of soils and marine sediments	Simultaneous run of large number of samples, low cost of equipment /Overestimation of organic matter content, not applicable to samples with carbonate and mineral matter
LECO carbon analysis Ref. [15]	Instrumental analysis, Quantitative method	TOC analysis in soils and sediments	Rapid, precise and reliable results /Expensive equipment
RockEval pyrolysis Ref. [16]	Instrumental analysis, Quantitative method	Hydrocarbon exploration in sediments, soil contamination	Fast sample preparation /Specialized method in hydrocarbon research
Catalytic oxidation method Ref. [20, 21]	Instrumental analysis, Quantitative method	TOC and TN analysis in soils and sediments	Simultaneous determination of TOC and TN, rapid, precise and reliable results, no production of hazardous wastes/Expensive equipment, Energy consumption for analysis

Concerning TOC analysis with the Wakley and Black wet oxidation-titration method, although it is low cost, it uses hazardous chemicals and underestimates samples with very low or high organic content. This can be easily concluded from Eq 1. For concentrations below 1% as the S value (mL used in blank titration) is very close to T value (mL used in sample titration) a slight misjudgement of the color change of the indicator near the end point can lead to a significant error. The same applies to the concentration range above 15% where the T value is lower than 1mL. This limitation of the wet oxidation-titration method was also found from the regression analysis of our results for TOC Low <1%, where R^2^ has the lowest value 0.825. As the TOC content was increasing the R^2^ was also increasing, (Table 3), indicating that the Wakley and Black method is satisfactory accurate for TOC concentrations around 5%.

Except of the above mentioned limitations, other restrictions of the Wakley and Black method resolved with the use of the catalytic oxidation method are: (i) possibility to measure colored samples where the color change of the indicator is difficult to be detected (ii) possibility to measure samples containing substances acting either as consumers or as inhibitors of the titration reaction (iii) significant reduce of chemical reagents.

Comparing the catalytic oxidation method with the other instrumental analytical methods presented in Table 4, we can see that NMR, hydrogen peroxide digestion and LOI are qualitative or semi-quantitative methods, while the qualitative RockEval pyrolysis is a specialized method in hydrocarbon research. The catalytic oxidation method, although it is an expensive method, gives fast precise and accurate results having the main advantage of the simultaneous determination of TOC and TN in soils and sediments.

## Conclusions

As there is a great demand from the scientific community and especially from geoscientists for reliable, and concurrent analysis of TOC and TN it is necessary to review and validate new methods and equipment. In this study, we reveal that the use of the high-temperature (720°C) catalytic (Pt/Al_2_O_3_) oxidation method, gives reliable results, rapidly and simultaneously. The comparison of TOC results of 206 samples (soils and marine sediments) measured with the catalytic oxidation method and the classical analysis of wet oxidation-titration yields a satisfactory regression line with a correlation coefficient of 0.985. The comparison of TN results of 43 of the above samples with elemental analysis also yields a regression line with a correlation coefficient of 0.977. The method validation, calculating precision and accuracy, level of detection, repeatability, and reproducibility, provides the proof that the method is suited for the analysis and fulfils the necessary quality requirements. The advantage of the method is that we can receive direct, simultaneous and accurate measurements of total organic carbon and total nitrogen for a variety of solid samples.

## Supporting information

S1 Table Data set of 206 samples for TOC regression analysis. (DOCX)

S2 Table Data set of 43 samples for TN regression analysis. (DOCX)

S1 Graphical abstract (DOCX)
