# Supplementary material for: Application of a catalytic oxidation method for the simultaneous determination of total organic carbon and total nitrogen in marine sediments and soils
Source: PLoS One. 2021 Jun 4;16(6):e0252308. doi: 10.1371/journal.pone.0252308 (PMC8177517; doi:10.1371/journal.pone.0252308)
Supplement: S1 Table — (DOCX) [file pone.0252308.s001.docx]

S1 Table. Data set of 206 samples for TOC regression analysis

| **Number of Samples** | **TOC (%) Wet oxidation** | **TOC (%) Catalytic Cobmustion Infrared** |
| --- | --- | --- |
| 1 | 2,26 | 2,81 |
| 2 | 1,13 | 1,19 |
| 3 | 0,92 | 0,97 |
| 4 | 0,32 | 0,33 |
| 5 | 0,16 | 0,19 |
| 6 | 1,74 | 1,98 |
| 7 | 0,38 | 0,39 |
| 8 | 0,64 | 0,66 |
| 9 | 3,94 | 3,69 |
| 10 | 0,85 | 0,93 |
| 11 | 0,74 | 0,79 |
| 12 | 0,64 | 0,72 |
| 13 | 0,59 | 0,60 |
| 14 | 0,53 | 0,63 |
| 15 | 1,11 | 1,20 |
| 16 | 0,31 | 0,39 |
| 17 | 0,69 | 0,83 |
| 18 | 0,48 | 0,40 |
| 19 | 0,40 | 0,47 |
| 20 | 0,70 | 0,77 |
| 21 | 0,90 | 1,09 |
| 22 | 0,91 | 0,84 |
| 23 | 0,86 | 0,94 |
| 24 | 0,95 | 1,03 |
| 25 | 0,71 | 0,78 |
| 26 | 0,85 | 0,96 |
| 27 | 0,96 | 0,97 |
| 28 | 0,42 | 0,48 |
| 29 | 0,42 | 0,49 |
| 30 | 0,47 | 0,52 |
| 31 | 0,52 | 0,55 |
| 32 | 0,71 | 0,80 |
| 33 | 0,91 | 0,97 |
| 34 | 0,81 | 1,22 |
| 35 | 0,47 | 0,67 |
| 36 | 2,33 | 2,35 |
| 37 | 2,52 | 2,67 |
| 38 | 3,03 | 3,04 |
| 39 | 1,48 | 1,50 |
| 40 | 1,87 | 1,94 |
| 41 | 1,35 | 1,37 |
| 42 | 2,72 | 2,92 |
| 43 | 1,91 | 2,02 |
| 44 | 2,90 | 3,08 |
| 45 | 1,94 | 2,06 |
| 46 | 2,48 | 2,61 |
| 47 | 2,08 | 2,25 |
| 48 | 2,33 | 2,51 |
| 49 | 2,53 | 2,33 |
| 50 | 0,60 | 0,72 |
| 51 | 3,01 | 2,92 |
| 52 | 1,02 | 1,11 |
| 53 | 3,27 | 3,57 |
| 54 | 1,23 | 1,32 |
| 55 | 1,07 | 1,05 |
| 56 | 1,09 | 0,97 |
| 57 | 2,68 | 1,92 |
| 58 | 0,28 | 0,27 |
| 59 | 1,70 | 1,01 |
| 60 | 0,67 | 0,67 |
| 61 | 3,35 | 3,28 |
| 62 | 1,56 | 1,51 |
| 63 | 0,61 | 0,40 |
| 64 | 1,71 | 1,49 |
| 65 | 2,68 | 2,45 |
| 66 | 4,52 | 4,57 |
| 67 | 0,23 | 0,27 |
| 68 | 0,14 | 0,11 |
| 69 | 3,67 | 3,90 |
| 70 | 1,06 | 1,16 |
| 71 | 0,37 | 0,37 |
| 72 | 0,56 | 0,64 |
| 73 | 0,70 | 0,63 |
| 74 | 1,87 | 1,92 |
| 75 | 0,46 | 0,42 |
| 76 | 4,60 | 3,90 |
| 77 | 3,74 | 3,73 |
| 78 | 1,63 | 1,58 |
| 79 | 2,03 | 1,93 |
| 80 | 4,64 | 4,02 |
| 81 | 1,18 | 1,24 |
| 82 | 0,89 | 0,86 |
| 83 | 1,59 | 1,50 |
| 84 | 1,61 | 1,53 |
| 85 | 1,06 | 1,04 |
| 86 | 1,65 | 1,63 |
| 87 | 1,29 | 1,11 |
| 88 | 1,57 | 1,50 |
| 89 | 0,92 | 1,00 |
| 90 | 0,74 | 0,68 |
| 91 | 0,46 | 0,43 |
| 92 | 4,83 | 4,49 |
| 93 | 1,65 | 1,01 |
| 94 | 1,16 | 0,91 |
| 95 | 1,89 | 1,41 |
| 96 | 0,98 | 0,91 |
| 97 | 0,85 | 0,78 |
| 98 | 0,57 | 0,51 |
| 99 | 3,84 | 3,36 |
| 100 | 0,93 | 0,65 |
| 101 | 0,65 | 0,63 |
| 102 | 0,38 | 0,49 |
| 103 | 0,76 | 0,66 |
| 104 | 0,71 | 0,52 |
| 105 | 0,37 | 0,37 |
| 106 | 0,52 | 0,40 |
| 107 | 0,47 | 0,44 |
| 108 | 0,42 | 0,42 |
| 109 | 0,56 | 0,64 |
| 110 | 1,16 | 1,01 |
| 111 | 1,08 | 0,83 |
| 112 | 0,37 | 0,37 |
| 113 | 0,33 | 0,30 |
| 114 | 0,42 | 0,49 |
| 115 | 3,33 | 3,09 |
| 116 | 2,65 | 2,08 |
| 117 | 1,56 | 1,70 |
| 118 | 0,71 | 0,76 |
| 119 | 0,61 | 0,54 |
| 120 | 0,40 | 0,43 |
| 121 | 0,23 | 0,34 |
| 122 | 0,84 | 0,75 |
| 123 | 0,70 | 0,58 |
| 124 | 0,88 | 0,59 |
| 125 | 0,51 | 0,36 |
| 126 | 0,74 | 0,68 |
| 127 | 0,93 | 0,86 |
| 128 | 0,79 | 0,61 |
| 129 | 0,65 | 0,58 |
| 130 | 0,82 | 0,75 |
| 131 | 0,87 | 0,79 |
| 132 | 0,90 | 0,86 |
| 133 | 0,94 | 0,91 |
| 134 | 0,67 | 0,51 |
| 135 | 1,04 | 0,97 |
| 136 | 0,66 | 0,64 |
| 137 | 4,51 | 4,68 |
| 138 | 4,44 | 4,76 |
| 139 | 9,49 | 13,21 |
| 140 | 8,23 | 10,02 |
| 141 | 7,96 | 8.76 |
| 142 | 6,48 | 7,12 |
| 143 | 14,75 | 17,32 |
| 144 | 12,20 | 13,39 |
| 145 | 9,13 | 9,97 |
| 146 | 30,23 | 40,77 |
| 147 | 29,13 | 34,39 |
| 148 | 32,13 | 42,49 |
| 149 | 18,59 | 23,71 |
| 150 | 24,18 | 33,48 |
| 151 | 32,27 | 36,25 |
| 152 | 15,95 | 22,88 |
| 153 | 9,45 | 14,81 |
| 154 | 9,56 | 13,42 |
| 155 | 5,52 | 6,44 |
| 156 | 3,78 | 4,05 |
| 157 | 3,85 | 4,13 |
| 158 | 4,51 | 4,68 |
| 159 | 4,33 | 4,84 |
| 160 | 4,23 | 3,84 |
| 161 | 4,60 | 4,11 |
| 162 | 4,37 | 4,09 |
| 163 | 4,47 | 4,26 |
| 164 | 5,21 | 4,92 |
| 165 | 4,23 | 4,38 |
| 166 | 4,60 | 4,66 |
| 167 | 4,37 | 4,61 |
| 168 | 4,11 | 4,45 |
| 169 | 5,33 | 5,05 |
| 170 | 5,89 | 5,24 |
| 171 | 3,06 | 2,94 |
| 172 | 3,11 | 2,99 |
| 173 | 3,45 | 3,33 |
| 174 | 3,36 | 3,24 |
| 175 | 2,97 | 2,87 |
| 176 | 2,42 | 2,33 |
| 177 | 2,41 | 2,36 |
| 178 | 2,85 | 2,87 |
| 179 | 3,85 | 3,79 |
| 180 | 2,83 | 2,72 |
| 181 | 3,25 | 3,23 |
| 182 | 3,99 | 3,98 |
| 183 | 3,11 | 2,99 |
| 184 | 3,14 | 3,06 |
| 185 | 3,92 | 3,89 |
| 186 | 3,89 | 3,84 |
| 187 | 3,36 | 3,24 |
| 188 | 2,75 | 2,72 |
| 189 | 2,71 | 2,65 |
| 190 | 1,51 | 1,48 |
| 191 | 3,93 | 3,88 |
| 192 | 2,23 | 2,04 |
| 193 | 2,73 | 2,66 |
| 194 | 3,06 | 2,89 |
| 195 | 2,34 | 2,24 |
| 196 | 2,13 | 2,04 |
| 197 | 3,40 | 3,19 |
| 198 | 3,93 | 3,84 |
| 199 | 0,44 | 0,39 |
| 200 | 2,63 | 2,66 |
| 201 | 2,99 | 3,06 |
| 202 | 3,24 | 3,20 |
| 203 | 2,10 | 2,05 |
| 204 | 3,60 | 3,19 |
| 205 | 3,08 | 3,23 |
| 206 | 3,27 | 3,07 |
