# Supplementary material for: Application of a catalytic oxidation method for the simultaneous determination of total organic carbon and total nitrogen in marine sediments and soils
Source: PLoS One. 2021 Jun 4;16(6):e0252308. doi: 10.1371/journal.pone.0252308 (PMC8177517; doi:10.1371/journal.pone.0252308)
Supplement: S2 Table — (DOCX) [file pone.0252308.s002.docx]

S2 Table. Data set of 43 samples for TN regression analysis

| **Number of Samples** | **TN (%) CHN Elemental Analyser** | **TN (%) Catalytic Combustion Chemiluminescence Method** |
| --- | --- | --- |
| 1 | 0,240 | 0,240 |
| 2 | 0,210 | 0,210 |
| 3 | 0,170 | 0,160 |
| 4 | 0,160 | 0,160 |
| 5 | 0,210 | 0,180 |
| 6 | 0,280 | 0,250 |
| 7 | 0,130 | 0,250 |
| 8 | 0,070 | 0,280 |
| 9 | 0,250 | 0,210 |
| 10 | 0,050 | 0,040 |
| 11 | 0,040 | 0,036 |
| 12 | 0,020 | 0,017 |
| 13 | 0,020 | 0,000 |
| 14 | 0,170 | 0,090 |
| 15 | 0,070 | 0,054 |
| 16 | 0,060 | 0,050 |
| 17 | 0,065 | 0,051 |
| 18 | 0,050 | 0,050 |
| 19 | 0,075 | 0,053 |
| 20 | 0,060 | 0,000 |
| 21 | 0,060 | 0,023 |
| 22 | 0,050 | 0,013 |
| 23 | 0,050 | <DL |
| 24 | 0,050 | 0,027 |
| 25 | 0,060 | 0,051 |
| 26 | 0,050 | 0,030 |
| 27 | 0,050 | <DL |
| 28 | 0,050 | 0,011 |
| 29 | 0,020 | <DL |
| 30 | 0,120 | <DL |
| 31 | 0,050 | <DL |
| 32 | 0,223 | 0,226 |
| 33 | 1,380 | 1,314 |
| 34 | 1,560 | 1,432 |
| 35 | 0,980 | 1,100 |
| 36 | 0,842 | 0,817 |
| 37 | 0,598 | 0,565 |
| 38 | 0,845 | 0,825 |
| 39 | 1,130 | 1,171 |
| 40 | 6,340 | 6,306 |
| 41 | 0,567 | 0,500 |
| 42 | 0,476 | 0,427 |
| 43 | 0,348 | 0,353 |
