# Supplementary figures and images for: Application of a catalytic oxidation method for the simultaneous determination of total organic carbon and total nitrogen in marine sediments and soils
Source: PLoS One. 2021 Jun 4;16(6):e0252308. doi: 10.1371/journal.pone.0252308 (PMC8177517; doi:10.1371/journal.pone.0252308)

S1 Graphical abstract


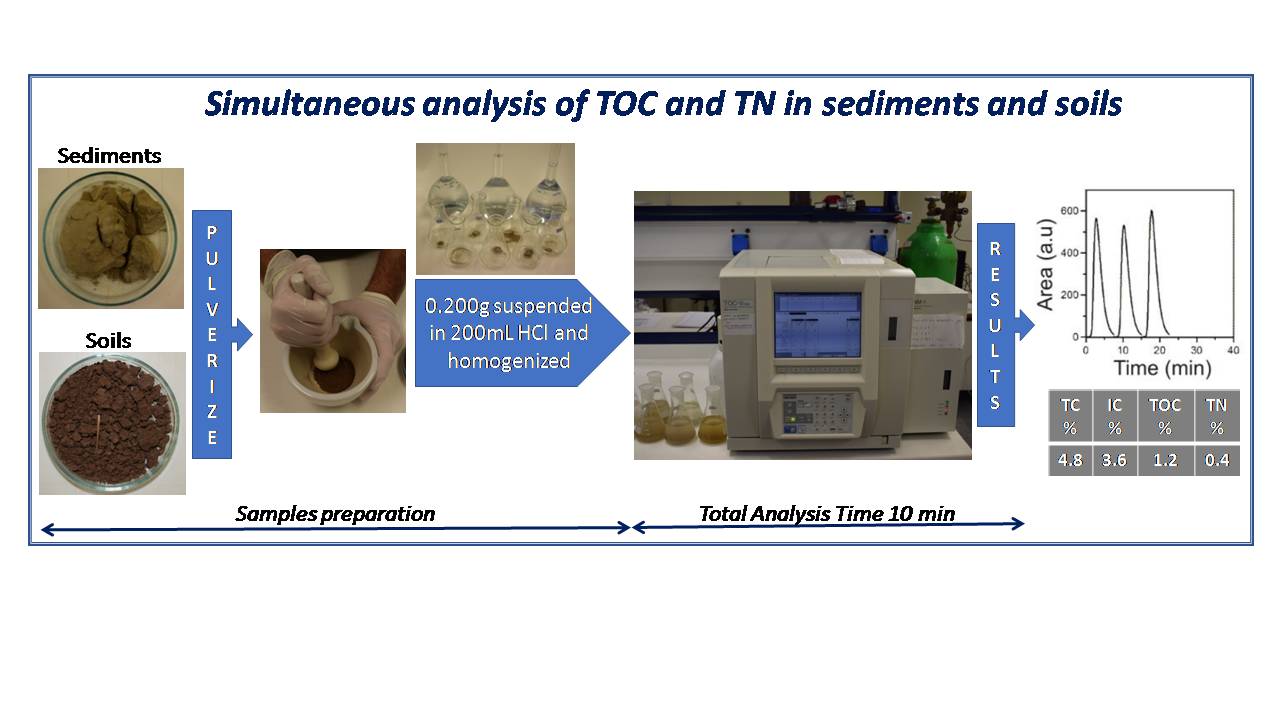

Supplement: S1 Graphical abstract — (DOCX) [file pone.0252308.s003.docx]
